# Predictive model for bacterial co-infection in patients hospitalized for COVID-19: a multicenter observational cohort study

**DOI:** 10.1007/s15010-022-01801-2

**Published:** 2022-04-29

**Authors:** Maddalena Giannella, Matteo Rinaldi, Giulia Tesini, Mena Gallo, Veronica Cipriani, Oana Vatamanu, Caterina Campoli, Alice Toschi, Giuseppe Ferraro, Clara Solera Horna, Michele Bartoletti, Simone Ambretti, Francesco Violante, Pierluigi Viale, Stefania Curti

**Affiliations:** 1 Infectious Diseases Unit, IRCCS Azienda Ospedaliero-Universitaria di Bologna, Department of Medical and Surgical Sciences, University of Bologna, Via Massarenti 11, 40137 SantBologna, Italy; 2 Department of Medical and Surgical Sciences, University of Bologna, Bologna, Italy; 3 Microbiology Unit, IRCCS Policlinico Sant’Orsola, Bologna, Italy; 4 Occupational Medicine Unit, Department of Medical and Surgical Sciences, IRCCS Policlinico Sant’Orsola, University of Bologna, Bologna, Italy

**Keywords:** SARS-CoV-2, COVID-19, Bacterial co-infection

## Abstract

**Objective:**

The aim of our study was to build a predictive model able to stratify the risk of bacterial co-infection at hospitalization in patients with COVID-19.

**Methods:**

Multicenter observational study of adult patients hospitalized from February to December 2020 with confirmed COVID-19 diagnosis. Endpoint was microbiologically documented bacterial co-infection diagnosed within 72 h from hospitalization. The cohort was randomly split into derivation and validation cohort. To investigate risk factors for co-infection univariable and multivariable logistic regression analyses were performed. Predictive risk score was obtained assigning a point value corresponding to β-coefficients to the variables in the multivariable model. ROC analysis in the validation cohort was used to estimate prediction accuracy.

**Results:**

Overall, 1733 patients were analyzed: 61.4% males, median age 69 years (IQR 57–80), median Charlson 3 (IQR 2–6). Co-infection was diagnosed in 110 (6.3%) patients. Empirical antibiotics were started in 64.2 and 59.5% of patients with and without co-infection (*p* = 0.35). At multivariable analysis in the derivation cohort: WBC ≥ 7.7/mm^3^, PCT ≥ 0.2 ng/mL, and Charlson index ≥ 5 were risk factors for bacterial co-infection. A point was assigned to each variable obtaining a predictive score ranging from 0 to 5. In the validation cohort, ROC analysis showed AUC of 0.83 (95%CI 0.75–0.90). The optimal cut-point was ≥2 with sensitivity 70.0%, specificity 75.9%, positive predictive value 16.0% and negative predictive value 97.5%. According to individual risk score, patients were classified at low (point 0), intermediate (point 1), and high risk (point ≥ 2). CURB-65 ≥ 2 was further proposed to identify patients at intermediate risk who would benefit from early antibiotic coverage.

**Conclusions:**

Our score may be useful in stratifying bacterial co-infection risk in COVID-19 hospitalized patients, optimizing diagnostic testing and antibiotic use.

**Supplementary Information:**

The online version contains supplementary material available at 10.1007/s15010-022-01801-2.

## Introduction

Bacterial co-infections have been associated with a huge increase in patient morbidity and mortality during influenza pandemics [1]. Such experience has prompted physicians to start empiric antibiotic coverage in patients hospitalized with COVID-19 [2]. In addition, several guidelines have recommended to initiate antibiotics within 1–4 h from hospital admission in patients with COVID-19 suspected of having bacterial co-infection and/or with criteria of sepsis or septic shock [3]; https://www.who.int/publications/i/item/clinical-management-of-covid-19; https://www.nice.org.uk/guidance/ng173).

Current literature shows that the rate of bacterial co-infection at hospital admission in patients with COVID-19 ranged from 3.5 to 11.6%, increasing to 14% among those with severe/critical disease who required intensive care [4–6]. However, more than two third of hospitalized patients were started on antibiotics [5–7]. Indeed, pre–post cross-sectional studies have revealed significant increase in antibiotic use during the first wave of COVID-19 pandemic compared with prior periods in their hospitals [8, 9]. In some cases, this finding has been associated with a concerning decrease in antibiotic susceptibility to several antibiotic classes among nosocomial pathogens such as *Klebsiella pneumoniae* [8]. Other reports have underlined an increased isolation of *Enterococcus* spp. from hospitalized patients with complicated COVID-19 course, probably because of antibiotic selective pressure [10].

Experts have exhorted physicians to not neglect antimicrobial stewardship principles during COVID-19 pandemic to avoid a worsening of the healthcare crisis related to antimicrobial resistance [11]. Thus, physicians are challenged to differentiate COVID-19 patients who may benefit from a prompt antibiotic therapy from those at low risk for co-infection where antibiotic pressure should be avoided. Studies performed to date have underlined a potential role in such discrimination process of biomarkers such as C-reactive protein (CRP), procalcitonin (PCT) and whole blood counts [12–15]. However, for all of them, low specificity with limited positive predictive value has been reported. Few authors have addressed the predictive role of clinical factors suggesting a role for steroid use, but in such studies also, superinfections were considered [15, 16].

Therefore, the objective of our study was to investigate the risk factors for bacterial co-infection at hospital admission for COVID-19 to create a predictive model able to stratify patients according to their probability of having a community-onset bacterial co-infection useful to guide microbiological work up and antibiotic use.

## Methods

### Study design

Multicenter observational study of patients hospitalized from February 22 through December 31, 2020, and diagnosed with COVID-19.

Diagnostic testing for COVID-19 and for bacterial co-infection were dictated by local policies and clinical judgement, and were not encompassed by a general protocol.

Clinical charts and hospital electronic records were used as data sources. Pseudo-anonymous data were prospectively collected and managed using REDCap electronic data capture tool, hosted by Alma Mater University of Bologna [17, 18].

The study was approved by the Ethic Committee of the promoting center (Comitato Etico Indipendente di Area Vasta Emilia Centro, n. 283/2020/Oss/AOUBo). Informed consent was obtained contacting patients via email or phone call. In case of deceased or unreachable patients, the informed consent was waived considering the observational nature of the study.

### Setting

The study was carried out at the three main hospitals of the metropolitan area of Bologna: (i) S. Orsola-Malpighi Hospital (1420-bed tertiary teaching hospital); (ii) Bellaria Hospital (320-bed tertiary center); and (iii) Maggiore Hospital (870-bed tertiary hospital). A unique infectious disease unit as well as a unique microbiology laboratory serve all the hospitals of the metropolitan area of Bologna.

### Participants

All consecutive adult (≥18 years) patients hospitalized with confirmed COVID-19 diagnosis by real-time reverse transcription polymerase chain reaction (RT-PCR) on nasopharyngeal swabs (NPS) were included. Patients on palliative care and/or discharged within 72 h from hospital admission were excluded as well as patients who acquired COVID-19 during hospital admission. Patients were followed-up to hospital discharge; those with multiple hospitalizations were included only once.

### Variables and definitions

The endpoint variable was a microbiologically documented bacterial co-infection diagnosed within 72 h from hospital admission defined according to clinical guidelines [19–21]. All positive microbiological assays were independently revised by two investigators (MR, CC) to confirm the diagnosis of bacterial co-infection against such criteria; any disagreement was resolved by a third senior investigator (MG).

The exposure variables were assessed at hospital admission and included: age, sex, body mass index, underlying conditions such as hypertension, immunosuppression and those included in the Charlson comorbidity index [22]. COVID-19 variables included: (i) date and symptoms at onset; (ii) date and symptoms at hospitalization; (iii) vital signs, laboratory tests and radiological findings. Clinical severity at hospitalization was recorded according to SOFA and CURB-65 scores. The attempt to diagnose bacterial co-infection defined as one or more samples collected for this purpose, the type of samples collected, the assays performed and their results were also recorded.

### Sample size

Based on literature data, bacterial co-infection rate among hospitalized patients with COVID-19 was estimated to be on average 8% [4, 5]. According to this, we planned to enroll at least 1600 patients to obtain 100 events with α = 0.10 and power of 90% (two-tailed hypothesis test) [23].

### Statistical analysis

The overall cohort of COVID-19 patients was randomly split into 70% of the cohort (derivation) and 30% of the cohort (internal validation) [24]. Descriptive statistics were calculated for the overall cohort as well as for the derivation and validation cohort. Continuous variables were expressed as median and interquartile range (IQR) and compared using Mann–Whitney *U* test. The assumption of normality of the variables was tested through the skewness and kurtosis test for normality as well as visual inspections. Categorical variables were reported as counts and percentages and compared with Pearson’s chi-squared test or Fisher’s exact test, as appropriate.

To develop the risk score, the analyses were initially performed in the derivation cohort, while the validation cohort was used to validate the predictive model.

Complete case analysis was performed. To investigate risk factors for bacterial co-infection univariable and multivariable logistic regression analyses were performed (i.e. derivation cohort). Odds ratios (ORs) and their corresponding 95% confidence intervals (95% CIs) were estimated. The covariates to be included in the multivariable logistic regression models were selected through a backward stepwise selection strategy (*P* value for inclusion ≤ 0.1, *P* value for exclusion > 0.2). Variables were primarily entered according to clinically relevance and lack of collinearity. The full model included the following variables: Charlson index, white blood cells (WBC), procalcitonin (PCT), C-reactive protein (CRP), and hypertension. The reduced model finally retained: Charlson index, WBC, and PCT. WBC was entered as a binary variable on the basis of the median value of coinfected patients, while the cut-offs for the other variables were assigned according to Youden's criterion [25]. Overall goodness of fit was evaluated by Nagelkerke's R^2^ and Hosmer and Lemeshow goodness of fit. Discrimination of the model was assessed by receiver-operator characteristics (ROC) curve of the predicted probability and Somers' D.

To develop the predictive risk score, variables in the final multivariable logistic regression model (i.e. derivation cohort) were assigned a point value corresponding to *β* coefficients rounded up to the nearest whole number [26]. The risk score was then calculated by adding individual points and categorized into three classes of risk (i.e. low, intermediate, and high) according to tertiles distribution. To better discriminate the risk for bacterial co-infection at intermediate level, a value of CURB-65 ≥ 2 was applied [27]. An optimized cut-point was then assigned using the Youden's J statistic and performance characteristics at cut-point (i.e. sensitivity, specificity, positive and negative predictive values) were calculated. For internal validation, these findings were then applied to the validation cohort. Discrimination measures were calculated to assess the model’s performance on the validation dataset.

To take into account other applications in clinical setting (e.g. cancer patients) an alternative predictive risk score was developed considering age, COPD, diabetes, renal diseases, and immunosuppression in place of Charlson index along with WBC and PCT (see supplementary Tables 1–3 and supplementary Fig. 1). We performed statistical analyses using Stata 16.1 (Stata Corp., College Station, TX, USA). All statistical tests were two-sided and an alpha error of 0.05 was accepted.

## Results

The study cohort consisted of 1811 patients hospitalized during the study period and diagnosed with COVID-19; 78 patients were excluded because they were treated with palliative care (*n* = 12), died or were discharged within 72 h from hospital admission (*n* = 43), or COVID-19 was diagnosed after 72 h from hospital admission (*n* = 23). Thus, 1733 patients were analyzed: 61.4% were males, with median age of 69 years (IQR 57–80), and median Charlson index of 3 (IQR 2–6) (Table 1).

**Table 1:** Clinical and demographic characteristics of the derivation and validation cohort

	Overall (*n* = 1733)	Derivation cohort (*n* = 1213)	Validation cohort (*n* = 520)	*p*-value
Demographic data				
Age (years), median (IQR)	69 (57–80)	69 (58–80)	69 (56–79)	0.243
Sex (male)	1048 (61.4)	460 (38.6)	199 (38.7)	0.951
Comorbidities				
Hypertension	953 (55.9)	666 (56.0)	287 (55.7)	0.928
Myocardial infarction	174 (10.0)	122 (10.1)	52 (10.0)	0.971
Congestive heart failure	136 (7.8)	94 (7.8)	42 (8.1)	0.816
Peripheral vascular disease	142 (8.2)	110 (9.1)	32 (6.2)	0.043
COPD	237 (13.7)	165(13.6)	72 (13.9)	0.892
Dementia	192 (11.1)	143 (11.8)	49 (9.4)	0.150
Diabetes without organ damage	244 (14.1)	159 (13.1)	85 (16.4)	0.076
Moderate/severe renal disease	144 (8.3)	95 (7.8)	49 (9.4)	0.271
Diabetes with organ damage	69 (4.0)	57 (4.7)	12 (2.3)	0.020
Any tumors within 5 years	166 (9.6)	114 (9.4)	52 (10)	0.696
Moderate/severe liver disease	16 (0.9)	10 (0.8)	6 (1.2)	0.584
Obesity	318 (19.2)	214 (18.5)	104 (20.8)	0.274
BMI (kg/m^2^), median (IQR)	25.3 (23.1–29.0)	25.0 (23.3–28.7)	29.4 (23.0–26.0)	0.640
Immunosuppression	76 (4.4)	49 (4.1)	27 (5.3)	0.274
Charlson index, median (IQR)	3 (2–6)	3 (2–5)	3 (1–6)	0.217
Clinical conditions at hospitalization				
Time from symptom onset to hospital admission (days), median (IQR)	6 (2–8)	5 (2–8)	6 (2–9)	0.235
Body temperature ≥ 38°	603 (35.3)	425(35.5)	178(34.7)	0.744
Dyspnoea	884 (51.8)	628 (52.5)	256 (50.0)	0.342
MAP, median (IQR)	90 (83–99)	92 (83–100)	90 (83–97)	0.188
Pulse rate, median (IQR)	87 (76–98)	88 (79–100)	86 (76–98)	0.619
Respiratory rate, median (IQR)	20 (16–24)	20 (16–24)	20 (16–24)	0.995
PaO2/FiO2, median (IQR)	300 (252–348)	300 (252–350)	305 (252–348)	0.990
Needing of oxygen support	1128 (66.7)	797 (67.4)	331 (65.0)	0.337
qSOFA, median (IQR)	0 (0–1)	0 (0–1)	0 (0–1)	0.892
SOFA score, median (IQR)	2 (1–3)	2 (1–3)	2 (1–3)	0.714
CURB-65, median (IQR)	1 (0–2)	1 (0–2)	1 (0–2)	0.754
Positive radiography at admission	611 (38.2)	427 (38.1)	184 (38.6)	0.856
Respiratory failure within 72 h from hospital admission	578 (33.4)	393(32.4)	185 (35.6)	0.198
ICU admission within 72 h from hospital admission	193 (11.1)	128 (10.6)	65 (12.5)	0.238
Antibiotic coverage at admission	1002 (59.8)	699 (59.8)	303 (59.9)	0.973
Diagnostic work-out				
At least one test performed within 72 h from hospital admission	1279 (80.7)	883 (80.6)	396 (80.8)	0.934
Blood cultures performed	545 (34.6)	364 (33.5)	181 (37.1)	0.164
Broncho-alveolar lavage	46 (2.9)	40 (2.8)	16 (3.3)	0.572
Pneumococcal urinary antigen	847 (53.6)	584 (53.5)	263 (53.7)	0.957
* L. pneumophila* urinary antigen	842 (53.3)	583 (53.4)	259 (52.9)	0.831
* M. pneumonia* serology	435 (27.6)	300 (27.6)	135 (27.7)	0.979
* C. pneumonia* serology	375 (23.8)	251 (23.1)	124 (25.4)	0.313
Flu test	134 (8.5)	93 (8.6)	41 (8.4)	0.932
Urine culture	222 (14.1)	143 (13.2)	79 (16.2)	0.106
Outcome				
Severe respiratory failure	790 (46.8)	549 (46.6)	241 (47.3)	0.794
NIV	275 (16.3)	179 (15.2)	96 (18.9)	0.060
ICU admission	318 (18.8)	212 (17.9)	106 (20.8)	0.159
All-cause 90 days mortality	339 (19.6)	237 (19.5)	102 (19.6)	0.970
Time to severe respiratory failure from hospital admission (days), median (IQR)	1 (0–4)	1 (0–4)	0 (0–3)	0.318
Time to ICU admission from hospital admission (days), median (IQR)	2 (1–5)	2 (1–5)	2 (0–6)	0.884
Time to death from hospital admission (days), median (IQR)	11 (6–20)	11 (6–20)	12 (7–21)	0.361
Laboratory tests at hospitalization				
WBC (mm^3^), median (IQR)	6.00 (4.43–8.32)	5.99 (4.43–8.17)	6.11 (4.42–8.51)	0.223
Neutrophils (10^9^/L), median (IQR)	4.62 (3.13–7.00)	4.62 (3.15–6.88)	4.64 (3.10–7.21)	0.489
Lymphocytes (10^9^/L), median (IQR)	1.01 (0.71–1.40)	0.72 (1.02–1.37)	0.69 (1.00–1.45)	0.939
Hemoglobin (g/dL), median (IQR)	13.3 (11.9–14.6)	13.3 (11.9–14.5)	13.2 (11.8–14.6)	0.857
PLT (10^9^/L), median (IQR)	198 (151–257)	198 (152–256)	199 (150–259)	0.880
INR, median (IQR)	1.10 (1.04–1.17)	1.10 (1.04–1.17)	1.10 (1.04–1.18)	0.917
Creatinine (mg/dL), median (IQR)	0.94 (0.76–1.18)	0.93 (0.75–1.18)	0.95 (0.77–1.20)	0.314
Glucose (mg/dL), median (IQR)	113 (99–137)	114 (99–138)	113 (99–137)	0.718
Ferritine (ng/mL), median (IQR)	385 (194–730)	388 (190–728)	365 (208–764)	0.967
LDH (U/L), median (IQR)	287 (227–373)	288 (229–369)	285 (225–379)	0.713
D-Dimer (mg/L), median (IQR)	0.76 (0.46–1.45)	0.78 (0.46–1.44)	0.71 (0.46–1.51)	0.055
IL-6 (pg/mL), median (IQR)	30.6 (14.3–61.7)	31.8 (15.1–65.0)	28.1 (12.3–49.8)	0.045
CRP (mg/dL), median (IQR)	6.4 (2.4–11.9)	6.4 (2.4–11.9)	6.3 (2.3–12.0)	0.783
PCT (ng/mL), median (IQR)	0.1 (0.0–0.1)	0.1 (0.0–0.1)	0.1 (0.0–0.1)	0.898
Lactates (mmol/L), median (IQR)	0.8 (1.1–1.5)	0.8 (1.1–1.5)	0.8 (1.1–1.5)	0.575

*IQR* interquartile range, *COPD* chronic obstructive pulmonary disease, *BMI* body mass index, *MAP*, mean arterial pressure, *SOFA* sequential organ failure assessment, *CURB65* confusion urea, respiratory rate and blood pressure, *ICU* intense care unit, *CPE* carbapenem-resistant Enterobacteriaceae, *NIV* non-invasive ventilation, *WBC* white blood cells, *PLT* platelets, *INR* international normalized ratio, *LDH* lactate dehydrogenase, *IL-6* interleukine 6, *CRP* C-reactive protein, *PCT* procalcitonin

All values given are *n* (%) unless otherwise stated

Bacterial co-infection was diagnosed in 110 (6.3%) patients. The most common types of bacterial co-infections were community acquired pneumonia and urinary tract infection that were diagnosed in 46 and 43 patients, respectively. Bloodstream infection at hospital admission was diagnosed in 26 patients, in 13 cases were deemed as primary, in 7 as device-related and in 6 as secondary. The most common causative agents were *Streptococcus pneumoniae* (*n* = 32) and *Escherichia coli* (*n* = 31) (Table 2).

**Table 2:** Description of co-infections

Site of infection	CAP	UTI	BSI			Total
			Primary	Device-related	Secondary	
Epidemiology						
* S. pneumoniae*	32	0	0	0	0	32 (29.1)
* Enterobacteriaceae*	0	36	1	0	4	37 (33.6)
* E. coli*	0	29	1	0	2	31 (28.2)
* K. pneumoniae*	0	7	0	0	2	7 (6.4)
* S. aureus*	4	0	1	1	0	6 (5.5)
* P. aeruginosa*	0	4	0	2	1	6 (5.5)
* Enterococcus spp*	0	3	1	1	0	5 (4.5)
Other *Streptococci*	0	0	1	0	0	1 (0.9)
CoNS	0	0	8	3	0	11 (10)
* M. pneumoniae*	9	0	0	0	0	9 (8.2)
* L. pneumophyla*	1	0	0	0	0	1 (0.9)
Other	0	0	1	0	1	2 (1.8)
Total	46	43	13	7	6	110 (100)

Patients were randomly divided into derivation (*n* = 1213) and validation cohort (*n* = 520). The comparison between derivation and validation cohort in terms of clinical and demographic characteristics is reported in Table 1. The rate of co-infections is 6.0% (73/1213) and 7.1% (37/520) in the derivation and validation cohort, respectively.

Descriptive characteristics of COVID-19 patients with and without bacterial co-infection along with univariable analysis of risk factors are reported in Tables 3 and 4 for derivation and validation cohort, respectively.

**Table 3:** Descriptive and univariable analysis for co-infections among COVID-19 patients in the derivation cohort (*n* = 1213)

	Cases with available data	Co-infections (*n* = 73)	No co-infections (*n* = 1140)	OR (95% CI)
Demographic data				
Age (years), median (IQR)	1213	75 (66–83)	69 (57–80)	1.03 (1.00–1.04)^a^
Sex (male)	1193	32 (43.8)	428 (38.2)	1.26 (0.78–2.03)
Comorbidities				
Hypertension	1190	49 (70.0)	617 (55.1)	1.90 (1.13–3.21)
Myocardial infarction	1213	9 (12.3)	113 (9.9)	1.28 (0.62–2.64)
Congestive heart failure	1213	9 (12.3)	85 (7.5)	1.75 (0.84–3.63)
Peripheral vascular disease	1213	9 (12.3)	101 (8.9)	1.45 (0.70–3.00)
COPD	1213	17 (23.3)	148 (13)	2.03 (1.15–3.60)
Dementia	1213	16 (21.9)	127 (11.1)	2.24 (1.25 4.02)
Diabetes without organ damage	1213	15 (20.6)	144 (12.6)	1.79 (0.99–3.24)
Moderate/severe renal disease	1213	11 (15.1)	84 (7.4)	2.23 (1.13–4.40)
Diabetes with organ damage	1213	5 (6.9)	52 (4.6)	1.54 (0.60–3.98)
Any tumors within 5 years	1213	5 (6.9)	109 (9.6)	0.70 (0.27–1.76)
Moderate/severe liver disease	1213	2 (2.7)	8 (0.7)	3.99 (0.83–19.12)
Obesity	1157	16 (22.5)	198 (18.2)	1.30 (0.73–2.32)
BMI (kg/m^2^), median (IQR)	717	24.0 (22.0–31.1)	25.0 (23.5–28.5)	0.99 (0.93–1.07)^a^
Immunosuppression	1202	6 (8.2)	43 (3.8)	2.26 (0.93–5.50)
Charlson index, median (IQR)	1213	5 (3–7)	3 (2–5)	1.16 (1.07–1.26)^a^
Clinical conditions at hospitalization				
Time from symptom onset to hospital admission (days), median (IQR)	1188	4 (1–8)	5 (2–8)	1.00 (0.97–1.02)^a^
Body temperature ≥ 38°	1196	23 (31.5)	402 (35.8)	0.99 (0.54–1.81)
Dyspnea	1196	45 (61.6)	583 (51.9)	1.49 (0.92–2.42)
MAP, median (IQR)	1151	90 (77–97)	92 (83–100)	0.98 (0.96–1.00)^a^
Pulse rate, median (IQR)	1153	88 (78–100)	87 (76–98)	1.01 (1.00–1.02)^a^
Respiratory rate, median (IQR)	1101	20 (18–28)	20 (16–24)	1.04 (1.00–1.07)^a^
PaO2/FiO2, median (IQR)	1104	276 (233–330)	301 (252–352)	1.00 (0.99–1.00)^a^
Needing of oxygen support	1182	56 (76.7)	741 (66.8)	1.64 (0.94–2.86)
qSOFA, median (IQR)	1150	1 (0–1)	0 (0–1)	1.62 (1.20–2.20)^a^
SOFA score, median (IQR)	1207	3 (2–4)	2 (1–3)	1.26 (1.11–1.43)^a^
CURB-65, median (IQR)	1207	2 (1–2)	1 (0–2)	1.66 (1.34–2.06)^a^
Positive radiography at admission	1121	29 (41.4)	398 (37.9)	1.16 (0.71–1.90)
Respiratory failure within 72 h from hospital admission	1213	41 (56.2)	352 (30.9)	2.87 (1.78–4.63)
ICU admission within 72 h from hospital admission	1213	15 (20.6)	113 (9.9)	2.35 (1.29–4.28)
Antibiotic coverage at admission	1169	48 (65.8)	651 (59.4)	1.31 (0.80–2.16)
Laboratory tests at hospitalization				
WBC (${\mathrm{mm}}^{3}$), median (IQR)	1184	7.69 (5.12–11.61)	5.93 (4.38–8.07)	1.20 (1.12–1.28)^a^
Neutrophils (10^9^/L), median (IQR)	1161	6.26 (3.35–9.31)	4.54 (3.13–6.71)	1.00 (1.00–1.00)^a^
Lymphocytes (10^9^/L), median (IQR)	1166	0.91 (0.60–1.38)	1.02 (0.73–1.37)	1.00 (0.99–1.01)^a^
Hemoglobin (g/dL), median (IQR)	1178	12.6 (11.0–13.8)	13.4 (12.0–14.6)	0.84 (0.76–0.94)^a^
PLT (10^9^/L), median (IQR)	1174	191 (153–277)	199 (152–256)	1.00 (1.00 –1.00)^a^
INR, median (IQR)	1085	1.13 (1.07–1.31)	1.10 (1.04–1.16)	1.23 (0.97–1.57)^a^
Creatinine (mg/dL), median (IQR)	1176	0.96 (0.80–1.43)	0.93 (0.75–1.17)	1.13 (0.95–1.34)^a^
Glucose (mg/dL), median (IQR)	1059	116 (99–150)	113 (99–137)	1.00 (0.99–1.01)^a^
Ferritine (ng/mL), median (IQR)	719	515 (214–1183)	376 (190–721)	1.00 (1.00–1.00)^a^
LDH (U/L), median (IQR)	1033	315 (249–356)	287 (228–370)	1.00 (1.00–1.00)^a^
D-Dimer (mg/L), median (IQR)	405	1.15 (0.81–1.68)	0.77 (0.45–1.42)	0.95 (0.77–1.18)^a^
IL-6 (pg/mL), median (IQR)	675	54.1 (24.3–131.4)	30.7 (15.0–62.3)	1.00 (1.00–1.00)^a^
CRP (mg/dL), median (IQR)	1156	10.9 (4.3–16.4)	6.2 (2.3–11.4)	1.06 (1.03–1.08)^a^
PCT ≥ 0.2 (ng/mL)	1123	45 (24.2)	23 (2.5)	12.68 (7.44–21.61)
Lactates (mmol/L), median (IQR)	808	1.5 (1.1–2.0)	1.1 (0.8–1.5)	1.00 (0.97–1.03)^a^

*IQR* interquartile range, *COPD* chronic obstructive pulmonary disease, *BMI* body mass index, *MAP* mean arterial pressure, *SOFA* sequential organ failure assessment, *CURB65* confusion urea respiratory rate and blood pressure, *ICU* intense care unit, *WBC* white blood cells, *PLT* platelets, *INR* international normalized ratio, *LDH* lactate dehydrogenase, *IL-6* interleukine 6, *CRP* C-reactive protein, *PCT* procalcitonin

All values given are *n* (%) unless otherwise stated

^a^For each year, point or unit increase

**Table 4:** Descriptive and univariable analysis for co-infections among COVID-19 patients in the validation cohort (*n* = 520)

	Cases with available data	Co-infections (*n* = 37)	No co-infections (*n* = 483)	OR (95% CI)
Demographic data				
Age (years), median (IQR)	520	78 (66–85)	68 (56–79)	1.02 (1.00–1.04)^a^
Sex (male)	514	16 (43.2)	183 (38.4)	1.22 (0.62–2.41)
Comorbidities				
Hypertension	515	26 (70.3)	261 (54.6)	1.97 (0.95–4.07)
Myocardial infarction	520	4 (10.8)	48 (9.9)	1.10 (0.37–3.23)
Congestive heart failure	520	4 (10.8)	38 (7.9)	1.42 (0.48–4.22)
Peripheral vascular disease	520	4 (10.8)	28 (5.8)	1.97 (0.65–5.95)
COPD	520	6 (16.2)	66 (13.7)	1.22 (0.49–3.04)
Dementia	520	10 (27.3)	39 (8.1)	4.22 (1.90–9.35)
Diabetes without organ damage	520	9 (24.3)	76 (15.7)	1.72 (0.78–3.79)
Moderate/severe renal disease	520	6 (16.2)	43 (8.9)	1.98 (0.78–5.01)
Diabetes with organ damage	520	1 (2.7)	11 (2.3)	1.19 (0.15 9.49)
Any tumors within 5 years	520	8 (21.6)	44 (9.1)	2.75 (1.19–6.39)
Moderate/severe liver disease	520	0 (0)	6 (1.24)	-
Obesity	500	3 (8.1)	101 (21.8)	0.32 (0.10 1.05)
BMI (kg/m^2^), median (IQR)	316	26 (23–27)	26 (23–30)	0.96 (0.86–1.07)^a^
Immunosuppression	513	6 (16.2)	21 (4.4)	4.19 (1.58–11.15)
Charlson index, median (IQR)	520	5 (4–7)	3 (1–5)	1.19 (1.07–1.32)^a^
Clinical conditions at hospitalization				
Time from symptom onset to hospital admission (days), median (IQR)	508	2 (0–6)	6 (2–9)	0.92 (0.86–0.98)^a^
Body temperature ≥ 38°	513	19 (51.4)	159 (33.4)	2.19 (0.96–4.99)
Dyspnea	512	20 (54.1)	236 (49.7)	1.19 (0.61–2.33)
MAP, median (IQR)	495	90.0 (83.3–96.7)	90.0 (83.3–96.7)	1.00 (0.97–1.03)^a^
Pulse rate, median (IQR)	492	90.0 (80–102)	85.0 (76–97)	1.02 (1.01–1.05)^a^
Respiratory rate, median (IQR)	459	20.0 (18–24)	20.0 (16–24)	1.01 (0.96–1.06)^a^
PaO2/FiO2, median (IQR)	488	289 (261–351)	305 (252–348)	1.00 (0.99–1.01)^a^
Needing of oxygen support	509	21 (60.0)	310 (65.4)	0.79 (0.39–1.60)
qSOFA, median (IQR)	497	0 (0–1)	0 (0–1)	1.11 (0.66–1.88)^a^
SOFA score, median (IQR)	517	2 (1–3)	2 (1–3)	1.17 (0.99–1.40)^a^
CURB-65, median (IQR)	517	2 (1–2)	1 (0–2)	1.88 (1.33–2.67)^a^
Positive radiography at admission	477	14 (37.8)	170 (38.6)	0.97 (0.48–1.93)
Respiratory failure within 72 h from hospital admission	520	13 (35.1)	172 (35.6)	0.98 (0.49–1.97)
ICU admission within 72 h from hospital admission	520	3 (8.1)	62 (12.8)	0.60 (0.18–2.01)
Antibiotic coverage at admission	506	20 (60.6)	283 (59.8)	1.03 (0.50–2.13)
Laboratory tests at hospitalization				
WBC (${\mathrm{mm}}^{3}$), median (IQR)	509	7.36 (5.51–10.24)	6.01 (4.37–8.51)	1.11 (1.01–1.22)^a^
Neutrophils (10^9^/L), median (IQR)	497	5.32 (3.69–8.29)	4.56 (3.08–7.2)	1.01 (0.96–1.06)^a^
Lymphocytes (10^9^/L), median (IQR)	501	0.80 (0.62–1.35)	1.00 (0.70–1.45)	0.62 (0.33–1.19)^a^
Hemoglobin (g/dL), median (IQR)	508	12.3 (10.7–13.7)	13.3 (11.9–14.7)	0.81 (0.69–0.94)^a^
PLT (10^9^/L), median (IQR)	506	222 (157–265)	197 (149–257)	1.00 (1.00–1.00)^a^
INR, median (IQR)	470	1.12 (1.04–1.19)	1.09 (1.04–1.18)	1.37 (0.55–3.45)^a^
Creatinine (mg/dL), median (IQR)	501	1.10 (0.79–1.58)	0.94 (0.77–1.18)	1.32 (1.06–1.65)^a^
Glucose (mg/dL), median (IQR)	452	110 (94–131)	114 (99–137)	1.01 (1.00–1.01)^a^
Ferritine (ng/mL), median (IQR)	303	439 (246–1167)	360 (202–753)	1.00 (1.00–1.00)^a^
LDH (U/L), median (IQR)	452	292 (221–432)	285 (226–376)	1.00 (1.00–1.00)^a^
D-Dimer (mg/L), median (IQR)	202	1.86 (0.67–2.29)	0.70 (0.46–1.40)	1.04 (0.96–1.13)^a^
IL-6 (pg/mL), median (IQR)	305	25.8 (12.9–48.5)	28.1 (12.3–49.8)	1.00 (0.99–1.01)^a^
CRP (mg/dL), median (IQR)	498	8.05 (2.76–14.16)	6.27 (2.31–11.7)	1.01 (0.97–1.05)^a^
PCT ≥ 0.2 (ng/mL)	489	20 (25.3)	10 (2.4)	13.56 (6.05–30.38)
Lactates (mmol/L), median (IQR)	356	1.1 (0.9–1.6)	1.1 (0.8–1.5)	0.99 (0.89–1.10)^a^

*IQR* interquartile range, *COPD* chronic obstructive pulmonary disease, *BMI* body mass index, *MAP* mean arterial pressure, *SOFA* sequential organ failure assessment, *CURB65* confusion urea respiratory rate and blood pressure, *ICU* intense care unit, *WBC* white blood cells, *PLT* platelets, *INR* international normalized ratio, *LDH* lactate dehydrogenase, *IL-6* interleukine 6, *CRP* C-reactive protein, *PCT* procalcitonin

All values given are *n* (%) unless otherwise stated

^a^For each year, point or unit increase

**Fig. 1:**
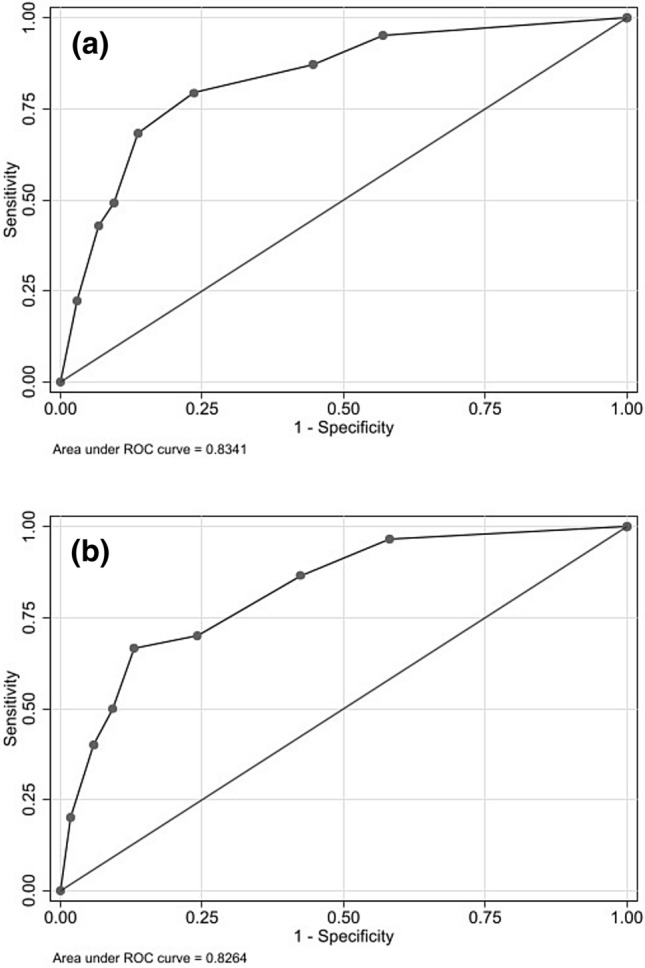
ROC curve in the derivation (**a**) and validation cohort (**b**)

At multivariable analysis in the derivation cohort: WBC ≥ 7.7/mm^3^, PCT ≥ 0.2 ng/mL, and Charlson index ≥ 5 were risk factors for bacterial co-infection and used for the predictive score (Table 5). For each variable a point was assigned according to the β coefficient rounded up to the nearest whole number. The predictive score resulted from the sum of the individual points and ranged from 0 to 5. In the ROC analysis, AUC was 0.83 (95%CI 0.78–0.88) (Fig. 1a) with Somers' D 0.67 (95%CI 0.57–0.77). Optimal cut-point was identified at risk score ≥ 2 with a sensitivity of 79.4% (95%CI 67.3–88.5%), specificity of 76.4% (95%CI 73.7–79.0%), positive predictive value of 17.2% (95%CI 13.0–22.0%) and negative predictive value of 98.4% (95%CI 97.2–99.1%).

**Table 5:** Multivariable logistic regression analysis for co-infections among COVID-19 patients and score development in the derivation cohort

	OR (95% CI)	*p*-value	*β*-coefficients	Points^a^
WBC (mm^3^)				
<7.70	Reference			0
≥7.70	1.63 (0.94–2.85)	0.083	0.49	1
PCT (ng/mL)				
<0.2	Reference			0
≥0.20	11.68 (6.62–20.61)	<0.001	2.46	3
Charlson Index				
<5	Reference			0
≥5	1.97 (1.12–3.44)	0.018	0.68	1

*WBC* white blood cells, *PCT* procalcitonin

^a^Point values were assigned according to β coefficients rounded up to the nearest whole number

In the validation cohort, the ROC analysis showed an AUC of 0.83 (95%CI 0.75–0.90) (Fig. 1b) with Somers’ D 0.65 (95%CI 0.51–0.80). At optimal cut-point of risk score ≥ 2, we found sensitivity of 70.0% (95%CI 50.6–85.3%), specificity of 75.9% (95%CI 71.7–79.7%), positive predictive value of 16.0% (95%CI 10.2–23.5%) and negative predictive value of 97.5% (95%CI 95.2–98.8%).

According to the suggested risk score, we classified patients at low, intermediate and high risk for bacterial co-infection, considering the value of CURB-65 ≥ 2 as a further element to discriminate patients at intermediate risk who would benefit from early antibiotic coverage (Table 6). The score applicability in terms of performance characteristics in the validation cohort is reported in Table 7. Using the score, 8 patients would be treated for each co-infection as compared to 16 that were treated without any score application.

**Table 6:** Suggested score interpretation

Scorea	Interpretation	Suggested management
0	Low risk	“Not to be treated”
1	Intermediate risk	If CURB65 < 2 “Not to be treated”
		If CURB65 ≥ 2 “To be treated”
≥2	High risk	“To be treated”

^a^Score is based on tertiles distribution. Score ranges from 0 to 5

**Table 7:** Score applicability in terms of performance characteristics in the validation cohort

	Low risk	Intermediate risk		High risk
Co-infections		Untreated	Treated	
No	191	93	62	110
	True negative = 284		False positive = 172	
Yes	1	4	4	21
	False negative = 5		True positive = 25	

Considering that the Charlson index could not be applicable in all settings (e.g. cancer patients), an alternative multivariable logistic regression model was developed using age and immunosuppression (in place of the Charlson index) along with WBC and PCT values. Data are shown in supplementary Tables 1–3 and supplementary Fig. 1.

## Discussion

We have investigated the rate of and the risk factors for bacterial co-infection, diagnosed within 72 h from hospital admission, in a large cohort of patients hospitalized for confirmed COVID-19 diagnosis. We developed a predictive risk score with few and easily accessible data (i.e. Charlson index, WBC and PCT) to stratify patients at low, intermediate and high risk of bacterial co-infection. For patients at intermediate risk, we propose to use CURB-65 severity score to further discriminate patients who may need early antibiotic coverage. The proposed score could be useful to standardize the approach to the microbiological work-up and to the therapeutic treatment of patients hospitalized for COVID-19 and suspected of having a bacterial co-infection.

As previously reported, we have shown a rate of bacterial co-infection lower than 10%, while the use of antibiotic therapy since the hospital admission was as high as 60% [4, 5]. It is worth mentioning that this rate was similar among patients with and without eventually confirmed bacterial co-infection to stress the difficulties in identifying among patients hospitalized with COVID-19 those with a concomitant bacterial co-infection as several symptoms and signs overlap between the SARS-COV2 infection and its complicated course and the bacterial disease [2, 28, 29]. For this reason, our risk score may be useful to standardize the approach to both the microbiological work-up, that could be avoid in patients at low risk at least in the most critical periods (i.e. pandemic waves, winter periods), and the therapeutic management reserving early antibiotic coverage only for patients at high risk. In addition, to optimize antibiotic use in patients at intermediate risk we propose to use the CURB-65, a well-known and largely used score for the management of patients with community-acquired pneumonia (CAP) [27, 30]. This allowed us to increase in the validation cohort the sensitivity of 13.3% points, and decrease the specificity of 13.6% points, as compared to baseline risk score (i.e. 70.0 and 75.9%, respectively).

Our study has several limitations. First, we limited our analysis only to microbiologically documented bacterial co-infection. Generally, in more than half of CAP the causative pathogens remain unknown despite an appropriate microbiological work-up. Thus, the prevalence of co-infection of the present study, mainly involving the respiratory tract, is likely to be underestimated, and consequently the PPV and NPV of our model may be biased. However, as the approach to clinical diagnosis and therapeutic management of bacterial (co-)infections is varying according to patient setting, clinical severity and physician specialty we have preferred to include only documented episodes to avoid the influence of several confounding factors in the definition of our events. Second, the approach to microbiological diagnosis of bacterial co-infection was not standardized during the study period, as it happened to the vast majority of centers during normal life [31] and even more during COVID-19 pandemic. Indeed, the overwhelmed emergency rooms, the overloading of microbiology laboratories, and the concerns for the safety of healthcare staff may have played a role in the performance and turnaround time of diagnostic testing for bacterial co-infections. However, we observed similar rates and types of bacterial co-infections described in literature suggesting a similar approach across centers dealing with high volume of COVID-19 hospitalizations to the microbiological diagnosis of bacterial co-infection in real life. Finally, the major drawback of randomly splitting the dataset into two parts (i.e. derivation and validation cohort) is that the precision of the fitted parameters will be reduced as only a part of the dataset is used for model derivation; this will also tend to give optimistic estimates of model performance [24]. To obtain reliable estimates, models should be externally validated as well.

To conclude, our study underlines that although the overall rate of bacterial co-infection among patients hospitalized with COVID-19 was lower than 10%, almost 60% of patients were started on antibiotics, and that the rate of antibiotic use was similar among patients with and without co-infection. Thus, our risk stratification in low, intermediate and high risk using a score based on Charlson index (alternatively on age and immunosuppression), WBC and PCT may be useful to guide diagnostic testing and to optimize antibiotic use. To improve its sensitivity in patients at intermediate risk, we also proposed to consider clinical severity using CURB-65, reserving antibiotic coverage only in patients with CURB-65 ≥ 2. External validation is needed to confirm the good performance of our score, and its impact on diagnostic and therapeutic management of hospitalized COVID-19 patients should be investigated.

## Supplementary Information

Below is the link to the electronic supplementary material.

Supplementary file1 (DOCX 98 KB)
