# Supplementary material for: Predictive model for bacterial co-infection in patients hospitalized for COVID-19: a multicenter observational cohort study
Source: Infection. 2022 Apr 29;50(5):1243–53. doi: 10.1007/s15010-022-01801-2 (PMC9053127; doi:10.1007/s15010-022-01801-2)
Supplement: Supplementary file 1 — Supplementary file1 (DOCX 98 KB) [file 15010_2022_1801_MOESM1_ESM.docx]

**Supplementary Table 1.** Multivariate logistic regression analysis for co-infections among COVID-19 patients and score development in the derivation cohort.

|  | **OR (95% CI)** | **p-value** | **β-coefficients** | **Points*** |
| --- | --- | --- | --- | --- |
| Age (years) |  |  |  |  |
| ≤70 | Reference |  |  | 0 |
| >70 | 1.53 (0.86-2.73) | 0.146 | 0.43 | 1 |
| WBC (mm^3^) |  |  |  |  |
| <7.70 | Reference |  |  | 0 |
| ≥7.70 | 1.75 (1.00-3.07) | 0.049 | 0.56 | 1 |
| PCT (ng/ml) |  |  |  |  |
| <0.2 | Reference |  |  | 0 |
| ≥0.2 | 12.01 (6.80-21.23) | <0.001 | 2.49 | 3 |
| Immunosuppression |  |  |  |  |
| No | Reference |  |  | 0 |
| Yes | 3.73 (1.33-10.45) | 0.012 | 1.32 | 2 |

Abbreviations: WBC, white blood cells; PCT, procalcitonin.

*Point values were assigned according to β coefficients rounded up to the nearest whole number.

**Supplementary Figure 1**. ROC Curve in the derivation (panel a) and validation cohort (panel b).

a)


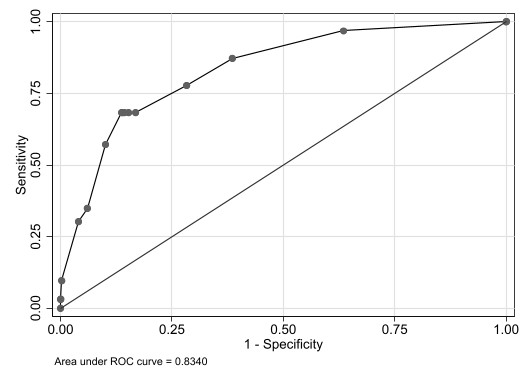


b)


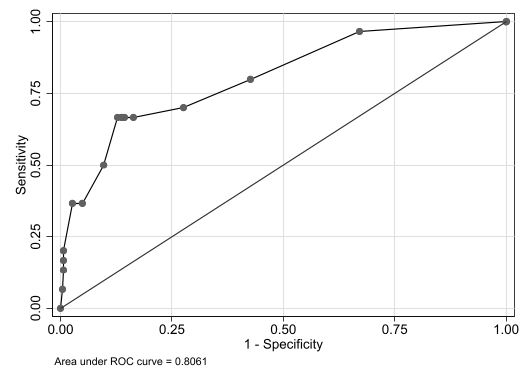


**Supplementary Table 2**. Suggested score interpretation.

| Score* | Interpretation | Suggested management |
| --- | --- | --- |
| 0 | Low risk | “Not to be treated” |
| 1 | Intermediate risk | If CURB65 <2 “Not to be treated” |
|  |  | If CURB65≥2“To be treated” |
| ≥2 | High risk | “To be treated” |

*The score is based on tertiles distribution. Score ranges from 0 to 7.

**Supplementary Table 3**. Score applicability in terms of performance characteristics in the validation cohort.

|  | Low risk | Intermediate risk | | High risk |
| --- | --- | --- | --- | --- |
| Co-infections |  | Untreated | Treated |  |
| No | 149 | 107 | 72 | 125 |
|  | True negative=256 | | False positive=197 | |
| Yes | 1 | 4 | 4 | 21 |
|  | False negative=5 | | True positive=25 | |
